# Vocal development in dystonic rats

**DOI:** 10.14814/phy2.12350

**Published:** 2015-04-23

**Authors:** Tobias Riede, Yu Zhao, Mark S LeDoux

**Affiliations:** 1 Department of Physiology, Midwestern UniversityGlendale, Arizona; 2 Department of Neurology, University of Tennessee Health Science CenterMemphis, Tennessee

**Keywords:** Bioacoustics, breathing, motor control, subglottal pressure

## Abstract

Vocal production, which requires the generation and integration of laryngeal and respiratory motor patterns, can be impaired in dystonia, a disorder believed due to dysfunction of sensorimotor pathways in the central nervous system. Herein, we analyze vocal and respiratory abnormalities in the dystonic (dt) rat, a well-characterized model of generalized dystonia. The dt rat is a recessive mutant with haploinsufficiency of *Atcay* which encodes the neuronally restricted protein caytaxin. Olivocerebellar functional abnormalities are central to the dt rat's truncal and appendicular dystonia and could also contribute to vocal and respiratory abnormalities in this model system. Differences in vocal repertoire composition were found between homozygote and wild-type dt rat pups developing after 3 weeks of life. Those spectro-temporal differences were not paralleled by differences in vocal activity or maximum lung pressures during quiet breathing and vocalization. However, breathing rhythm was slower in homozygote pups. This slower breathing rhythm persisted into adulthood. Given that cerebellectomy eliminates truncal and appendicular dystonia in the dt rat, we hypothesize that the altered breathing patterns stem either from a disturbance in the maturation of respiratory pattern generators or from deficient extracerebellar caytaxin expression affecting normal respiratory pattern generation. The altered breathing rhythm associated with vocal changes in the murine model resembles aspects of vocal dysfunction that are seen in humans with sporadic dystonia.

## Introduction

Our current understanding of normal vocal motor control and disorders of voice production in humans remains fragmentary. Normal voice production requires vocal fold posturing by intrinsic laryngeal muscles and breathing movements that build up lung pressure to generate air flow. Few nonhuman mammalian models are available for rigorous testing. Rodent ultrasound vocalization (‘USV’) serves as a phenotypic marker in models of neurodevelopmental and sociocognitive disorders (e.g., Brudzynski 2013). USVs can also serve as an indicator of vocal motor dysfunction because acoustic features are the result of the precise control of breathing and laryngeal movements (Roberts 1972; Hegoburu et al. 2011; Riede 2011, 2013, 2014). Furthermore, vocal behavior in rats is interdependently integrated with multiple orofacial sensorimotor behaviors such as whisking and breathing (e.g., Kim and Bao 2009; Rao et al. 2014; Sirotin et al. 2014). In order to determine to what extent vocal changes in rodent models of neurological disorders are based on altered vocal and respiratory motor patterns, we investigated the dystonic rat (‘dt rat’), an animal model of primary general dystonia (LeDoux 2011). This is the first study investigating underlying vocal motor patterns in an awake and spontaneously behaving animal model.

The dt rat is a spontaneous mutant discovered in the Sprague–Dawley strain (Lorden et al. 1984), with functional abnormalities in the olivocerebellar pathways (LeDoux et al. 1998; LeDoux 2005; Xiao et al. 2007). Homozygote pups develop a dystonic motor syndrome that visibly affects the truncal and appendicular musculature by postnatal day 12 (P12). The purpose of this study was to determine whether their vocal production was also affected. Since the cerebellum is critically involved in the modulation of respiratory motor patterns (e.g., Xu and Frazier 2000), and the production of USV is intimately linked to the respiratory system, vocal changes could result from altered respiratory control.

Dystonic rats harbor an insertional mutation in *Atcay* which encodes caytaxin, a protein which plays an important role in the postnatal maturation of cerebellar cortex (LeDoux et al. 1998; Xiao et al. 2007). Homozygote dt rat pups show axial and appendicular dystonia after P12. They will not survive past P35, unless the cerebellum is removed (LeDoux et al. 1993). We studied vocal behavior during the first 4 weeks of life, a period in which the onset of dystonic symptoms in homozygote dt rats overlaps with a significant growth spurt (5 g at birth to about 25 g at P15) and at least three neurophysiological maturation processes. Pups begin to hear, and auditory experiences have long-lasting impacts on adult brain sound representation starting at P10 (e.g., Geal-Dor et al. 1993; de Villers-Sidani et al. 2007), adult respiratory patterns crystallizes between P10 and P15 (Paton and Richter 1995; Dutschmann et al. 2009), and the ingestive behavior transforms from suckling to mastication of hard food starting on P12 (Iriki et al. 1988; Westneat and Hall 1992). All four processes can affect vocal production, some in a predictable way (larger vocal organs likely lower pitch), whereas others are not fully understood. In the dt rat, it is unknown whether or not there are deficits in vocal motor control.

## Methods

Pup vocalizations from ten litters were recorded. Each female with a litter was housed in a standard rodent cage. Respiratory patterns of adult cerebellectomized dt rats were compared with data from normal Sprague–Dawley rats of similar size and age. All rats were individually marked with a tattoo. Procedures involving animals and their care were reviewed and approved by the Institutional Animal Care and Use Committees (IACUC) of the University of Utah, Midwestern University and the University of Tennessee Health Science Center.

### Sound recording and acoustic analysis in pups

We recorded vocalizations from all pups from each litter. Calls from 15 homozygote (20630 calls) and 18 wild-type (20166 calls) pups were analyzed. Pups were genotyped at the end of the experiments. Vocal behavior was recorded on 8 days between P1 and P30. Recordings were pooled into one of eight bins: P1-2, P3-5, P6-8, P9-11, P12-14, P15-19, P20-24, and P25-30. Calling behavior was provoked while the pup was isolated from the litter for 5 min. The pup was placed in a round plastic container with a microphone positioned 15 cm above the container floor. For each recording time point, data for at least five pups were pooled. All recorded calls during the 5-min session were included unless signal-to-noise-ratio did not allow an automated analysis.

Sound was recorded by a condenser microphone (Avisoft-Bioacoustics CM16/CMPA-5V, Berlin, Germany), acquired through an NiDAQ 6212 acquisition device, sampled at 200 kHz, and saved as uncompressed files using Avisoft Recorder software (Avisoft-Bioacoustics).

Calling rates were determined by counting calls over the entire recording interval. Rates were expressed as “number of calls per second”. Call types were defined based on spectrographic appearance, following earlier descriptions (Brudzynski et al. 1999; Zeskind et al. 2011). Pup calls were differentiated into 11 call types according to call duration, fundamental frequency range, and presence of specific features such as frequency jumps or trill components (Table1).

**Table 1:** Call types in pups were assorted into one of 11 types using spectral and temporal features.

Call type	Spectro-temporal features
Fast down calls	10–20 ms, 30–60 kHz, <2 kHz downward frequency modulation
Multistep calls	50–200 ms, 40–60 kHz, bandwidth 2 to 4 kHz; one or more jumps; there is no pause at the jump
Complex calls	100–300 ms, 35–60 kHz, fast FM at rates between 60 and 100 Hz
Slow down calls	100–250 ms, 40–60 kHz, <3 kHz bandwidth
Meander calls	80–200 ms, 30–50 kHz, sharp up or down FM; unlike in step calls, the sudden F0 change is continuous
Flat calls	80–200 ms; 46–55 kHz; bandwidth <2 kHz
Slow up calls	20–80 ms, 30–60 kHz, <2 kHz upward FM
Multipart calls	50–200 ms, 40–60 kHz, bandwidth 2 to 4 kHz; one or more jump-like F0 change, but the transition is continuous
Short calls	Less than 20 ms in duration, 30–100 kHz, no apparent frequency modulation
Tipped S calls	80–200 ms, 35–60 kHz, up FM followed by down FM followed by down FM
Fast up calls	10–20 ms, 30–60 kHz, <2 kHz upward FM

Call duration and fundamental frequency were quantified using the frequency tracking tool of Avisoft-SAS-lab Pro software to investigate the acoustic structure of call types. Automated measurements excluded calls with insufficient signal-to-noise ratio. SAS-lab Pro allows the investigator to overlay spectrographic images and fundamental frequency tracking results. Thereby, the correct tracking was visually confirmed. We analyzed 40796 calls from 33 pups. Four parameters were compared between wild-type and homozygote pups: (1) call duration, (2) mean fundamental frequency [“mean F0”], (3) fundamental frequency at mid-call [“center F0”], and (4) range of the fundamental frequency calculated as the difference between maximum and minimum value [“F0-range”]. F0-range provides information about the modulation of fundamental frequency. In pups, for example, flat calls tend to have a much lower F0-range than meander calls. Investigations in adult rats showed that such features reflect muscle activity in the larynx and respiratory system (Riede 2013).

### Subglottal pressure recording

Subglottal pressure provides an indirect measure of the effort put into vocal production and is associated with the activity of muscles contributing to the build-up of expiratory pressure and laryngeal muscles that regulate the laryngeal valve.

Subglottal pressure was measured in four wild-type and four homozygote pups between P8 and P12 via a stainless steel tube (L-shaped, two branches of the tube are 3 and 4 mm in length; 0.5 mm inner diameter) implanted in the upper third of the trachea as previously described (Riede 2011). The stainless steel tube was connected to a pressure transducer (model FHM-02PGR-02; Fujikura Ltd, Tokyo, Japan) via a silastic tube. Rats wore a small custom-made jacket on which the pressure transducer was mounted and a tether connected. After the animals awoke and became fully ambulatory, they were returned to their home cage for up to 4 h at which point the experiment was terminated. The calibrated pressure signal was recorded simultaneously with the sound signal into a second channel of the acquisition board, using Avisoft Recorder software.

In order to test if aberrant breathing patterns found in homozygote pups persist into adulthood, we also performed tracheal pressure recordings in adult dt rats. Previous studies have shown that dt rats will not survive beyond P35, unless the cerebellum is removed (LeDoux et al. 1993). Therefore, cerebellectomies (CBX) were performed in 15 pups (five homozygous males, five heterozygous males, and five heterozygous females) on P15. Surgeries were done through a vertical incision on the scalp and suboccipital craniectomy to expose the cerebellum. The entire cerebellum, including the cerebellar nuclei was removed by subpial suction. The resultant cavity was filled with Avitene (Davol, Warwick, RI). Wounds were closed with absorbable suture and tissue adhesive. Upon completion of physiological studies, brains were removed and sectioned on a cryostat in the coronal plane. Brain sections were mounted and stained with cresyl violet to confirm the extent of CBXs. We were able to successfully record tracheal pressure for a period of 2–4 days from four homozygote and seven heterozygote CBX rats (250–300 g body weight, 4–5 months of age). Six intact adult Sprague–Dawley of same ages and sizes served as controls. Breathing cycle durations were analyzed over 30-min periods during which rats were located in their home cage resting, feeding, grooming, and vocalizing.

### Genotyping

To genotype dt rat pups, gDNA was extracted from tail snips and PCR amplified using a primer pair flanking the insertional mutation (435 bp) in Intron 1 of *Atcay* (forward: gacacaatggatttgactcagc, reverse: aggtctttactggctcagctct) yielding 697 bp and 1132 bp amplicons for wild-type *Atcay* and *Atcay*^dt^ alleles, respectively. An additional reverse primer targeting the insert (gcatgaactcccatttagcata) was used to generate a 488 bp amplicon in the presence of *Atcay*^dt^. PCR amplification was performed with Taq2000 (Stratagene, La Jolla, CA, www.stratagene.com) in the following manner: 94°C for 3 min, 35 cycles at 94°C for 30 sec, 58°C for 30 sec and 72°C for 45 sec, with final extension occurring at 72°C for 10 min.

### Statistics

Data are presented as mean and SEM. For all measured parameters, the mean from each rat were averaged so that only one value per individual, parameter and age were entered in statistical tests. The effects of genotype (wild-type vs. homozygote) and age (P2; P5; P8; P11; P14; P19; P24; P30) (independent variables) on acoustic parameters (call rate, call duration; mean F0/center F0/F0-range) (dependent variables) were investigated in one-way ANOVAs and subsequent Tukey tests. Differences in subglottal pressure between homozygote and wild-type animals were investigated with Mann–Whitney tests.

## Results

### Calling activities are similar

**Figure 1:**
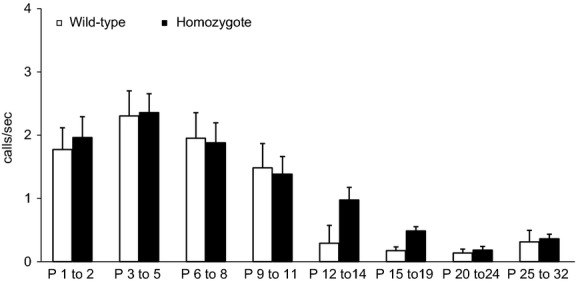
Development of vocal activity. Call rates of wild-type and homozygote dt rat pups. P, postnatal day. Rates increase to a maximum between day 3 and 5 before they decrease to almost zero in the third week of life. Data are means ± SEM.

Ontogenetic changes in vocal activity were not different between wild-type and homozygote animals (*F*_1,14_ = 0.11, *P* = 0.74). Between P3 and P5, call rates increased to 2.3 and 2.5 calls/sec, in wild-type and homozygote animals, respectively (Fig.1). The rates decreased to <1 call/s after P14. Calling rates changed with age in both groups (wild-type: *F*_7,54_ = 8.3, *P* < 0.01; homozygote: *F*_7,78_ = 8.0, *P* < 0.01).

### Call type rates differ

Flat calls were the most abundant call types in both groups followed by “tipped-S” calls on P2 and P5. The call distribution across eleven call types differed over time in homozygote (*F*_40,297_ = 1.8; *P* < 0.01) but not in wild-type pups (*F*_40,229_ = 1.1; *P* = 0.23), suggesting that the composition of the repertoires changed differently in the two groups. In wild-type pups, the flat calls became less abundant in favor of call types with modulated fundamental frequency contours such as meander, multi-step, multi-part, and complex calls. In wild-type pups, complex calls were first observed on P5, and in homozygote dt pups they appeared first on P8.

### Spectral acoustic parameters of USV differ

**Figure 2:**
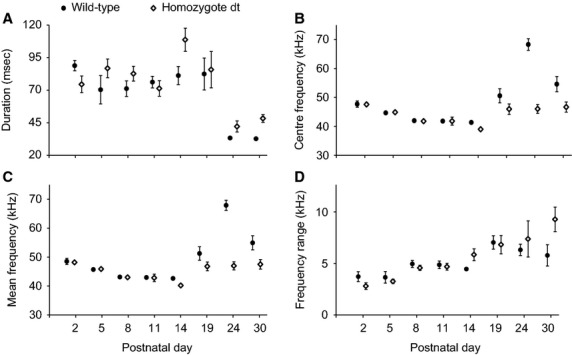
Spectro-temporal analysis of pup vocalizations. Four acoustic variables ((A) call duration, (B) center fundamental frequency, (C) mean fundamental frequency, (D) fundamental frequency range) were measured in ultrasonic calls from wild-type and homozygote dystonic rat pups. Isolation calls were recorded between P1 and P30. Data are means ± SEM. An individual pup contributed one data point to each mean since all its calls were pooled in each recording period. Wild-type and homozygote pups were sampled at the same age. Comparisons (one-way ANOVAs and subsequent Tukey tests) suggested that mean and center frequency as well as frequency range were different between wild-type and homozygote pups on P24 and P30.

Call duration showed no consistent differences with age and genotype (*F*_7,106_ = 1.15, *P* = 0.33), but center frequency (*F*_7,106_ = 10.9, *P* < 0.001), and mean frequency (*F*_7,106_ = 10.4, *P* < 0.001) were significantly lower and frequency range (*F*_7,106_ = 2.75, *P* < 0.012) were significantly larger in homozygote than in wild-type pups as they grew older. Figure2 shows development of averages for four acoustic parameters broken up for wild-type and homozygote pups during the first 4 weeks of life.

### Similar respiratory effort but different breathing rates

**Figure 3:**
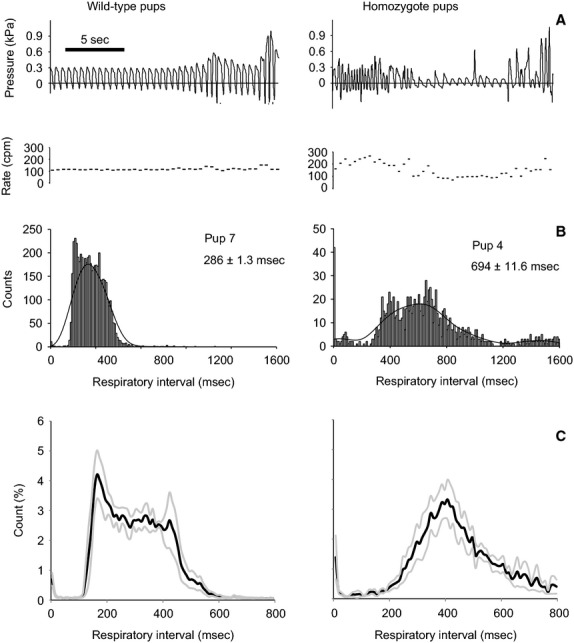
Different breathing patterns in wild-type and homozygote dt rat pups. A 20-sec record of breathing activity of a tethered pup in the home cage (A, top panel). Breathing rates (A, second panel) are based on breathing cycle durations and were calculated from a pressure signal recorded in the trachea. (B) Two example histograms of respiratory intervals recorded during 30 min of spontaneous behavior. Data are means ± SEM. (C) Average envelopes of breathing interval histograms (10 ms bin size) from four homozygote and four wild-type animals. Black lines are means, gray lines represent one SEM. Note the different scaling in B and C.

The duration of respiratory cycles was measured for 30 min of continuous recording during which the pups were in their normal environment. Mean respiratory cycle duration was significantly shorter in wild-type pups (300 ± 36 ms) than in homozygote pups (492 ± 136 ms) (*Z* = 2.3, *P* = 0.02) (Fig.3).

**Figure 4:**
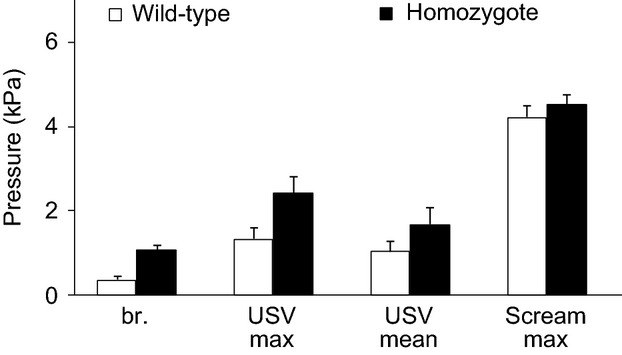
Similar vocal effort in normal and dt pups. Maximum subglottal pressure of wild-type and homozygote dt rat pups during breathing (br.), USV and audible vocalization (scream). In USV subglottal pressure was measured at the moment of maximum pressure (USV max) and averaged over the entire respiratory cycle during which a call is produced (USV mean). Data are means ± SEM.

These differences in breathing pattern appear not to be caused by lack of motor power because mean peak subglottal pressures were slightly higher in homozygote pups but not statistically significant during various tasks (normal respiration, USV and scream production) (Fig.4). Peak pressure ranged between 0.2 and 0.6 kPa during normal respiration in wild-type pups, and between 0.3 and 1.1 kPa in dt animals (Mann–Whitney tests, *N*_1,2_ = 4; *Z* = 1.73, *P* = 0.08). Mean subglottal pressure during USV was 1.4 kPa and 1.6 kPa in wild-type and homozygote pups, respectively (*Z* = 1.41, *P* = 0.15). Peak subglottal pressures during USV were 1.7 and 2.3 kPa, respectively (*Z* = 1.06, *P* = 0.28). During audible vocalizations, subglottal pressure often exceeded values measured during USV. Mean subglottal pressure was 2.7 kPa and 3.0 kPa in wild-type and homozygote pups, respectively (*Z* = 0.86, *P* = 0.38), and maxima reached 4.3 and 4.6 kPa, respectively (*Z* = 0.86, *P* = 0.38).

### Breathing patterns in dt rats remain affected into adulthood

**Figure 5:**
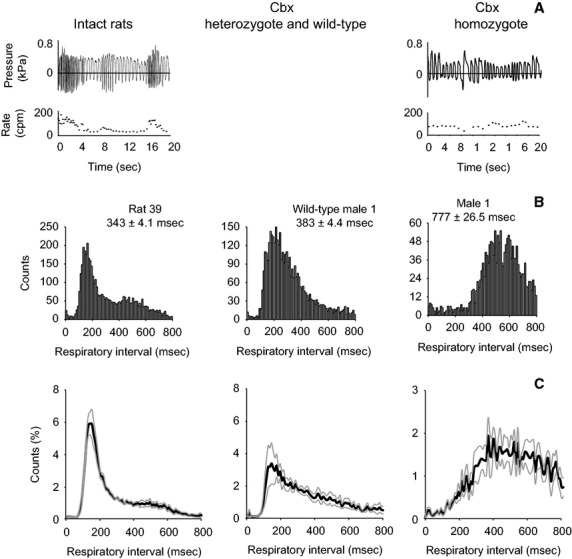
Different breathing patterns in wild-type and homozygote dt adult rats. (A) A 20-sec record of breathing activity (subglottal pressure and breathing rate in cpm). (B) Three examples of histograms of respiratory intervals recorded during 30 min of spontaneous behavior (resting and exploratory behavior in home cage), from a homozygote and a heterozygote CBX male, and from an intact wild-type male. The breathing intervals were combined into 10 ms bins. (mean ± SEM) (C) Average envelopes of breathing interval histograms from four homozygote CBX animals, seven heterozygote CBX animals and six intact wild-type animals. (dark line is mean, gray lines represent SEM).

Homozygote pups survive into adulthood if the cerebellum is removed. In 11 cerebellectomized adult dt rats (four homozygotes and seven heterozygotes) and in six intact control animals, subglottal pressure was measured during quiet breathing, 20 kHz USV, 50 kHz USV, and audible vocalizations. On average, breathing cycle durations were longer in homozygote dt rats than in heterozygote CBX rats (*F* = 16.4, *P* < 0.01) or in intact animals (*F* = 16.2, *P* < 0.01), but there was no difference between heterozygote CBX rats and intact rats (*F* = 0.31, *P* = 0.59) (Fig.5). The fast breathing cycle component (“sniffing”; respiration interval between 80 and 150 ms) was found less frequently in homozygote dt rats. The ratio of expiratory and inspiratory cycle duration was not different between homozygote and heterozygote CBX rats (*Z* = −0.9, *P* = 0.35), or between CBX rats and normal rats (*Z* = 0, *P* = 1), suggesting that the slower breathing rhythm in homozygotes is not caused by a more elaborate breathing pattern such as one due to a greater laryngeal resistance during one phase of the breathing cycle. Breathing in the four homozygote males was also characterized by episodes of arrhythmic breathing and extended periods with increased subglottal pressure. For example, there were long periods during exacerbation of dystonic movements (i.e., “dystonic attacks or storms”). Dystonic attacks were characterized by extreme torsion of the trunk and neck and extension of hindlimbs. During these paroxysmal episodes which lasted up to 20 sec, the animal would hold its breath with subglottal pressure raised to normal expiratory pressure.

**Figure 6:**
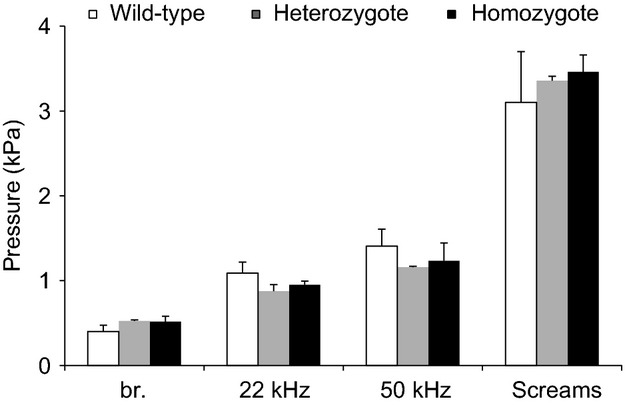
Similar vocal effort in normal and dt adult rats. Maximum subglottal pressure measured in six intact rats and in eleven CBX rats (four homozygotes, seven heterozygotes) during breathing (br.), 22 kHz USV, 50 kHz USV, and audible vocalization (screams). Data are means ± SEM.

Maximum subglottal pressure was compared during quiet breathing, 20 kHz, 50 kHz USV, and audible vocalizations (Fig.6). No significant differences were found on any parameter. Average maximum expiratory pressure during quiet breathing ranged between 0.4 and 0.6 kPa (*Z* = 0.38, *P* = 0.69); during 22 kHz calls between 0.7 and 1.1 kPa (*Z* = 0.46, *P* = 0.64), and during 50 kHz calls between 0.95 kPa and 1.5 kPa (*Z* = 0.38, *P* = 0.69). Maximum subglottal pressure during audible vocalizations ranged between 2.6 kPa and 3.5 kPa.

## Discussion

The results of this study provide three findings that contribute to our understanding of vocal control in a dystonic rodent model. First, spectral and temporal acoustic differences in USV between wild-type and homozygote dt rat pups were initially inconsistent but became more prominent after P14. Most striking is the divergent development of breathing patterns associated with these vocal differences. Average breathing rates in heterozygote and wild-type pups (300 ms or ∼3 Hz) correspond well with published data (e.g., Zehendner et al. 2013), however breathing rhythm in homozygote pups was different. This study showed also that the fast breathing component (“sniffing”; 6 to 8 Hz breathing rate) occurs much less frequently in homozygote adults, than in heterozygote or wild-type animals. The wide distribution of breathing rates in wild-type adult rats (sniffing: 6 to 8 Hz; and quiet breathing: 2 to 3 Hz) has been described before (e.g., Haston et al. 1993; Gray et al. 2001). The relationship between breathing rates and acoustic features of USV has been addressed in studies demonstrating that call type features are tightly related to underlying motor patterns (Roberts 1972; Riede 2013) and that rat USV are often embedded in fast (8 Hz) breathing episodes (Rao et al. 2014; Sirotin et al. 2014). Since overall call rates did not diminish but average breathing rates decreased, this suggests a causal dependency of call production on breathing dynamics on a fine time scale (e.g., acceleration of flow rate and/or lung pressure build up) rather than on a coarse time scale (e.g., average breathing rate). Rat USV are produced by a whistle mechanism (Roberts 1975; Riede 2011). A whistle is characterized by the conversion of air flow energy to sound by an interaction between the laryngeal configuration and the air stream. This fluid mechanical interaction is highly dependent on flow rate and the rate at which pressure builds up (Wilson et al. 1970). Although the different breathing patterns are very prominent, we cannot completely rule out that differences of acoustic parameters between homozygote and wild-type animal are in part caused by differential growth of the vocal organ, maturation of the respiratory system, or development of brain stem vocal pattern generators. Furthermore, the contributions of laryngeal coordination to this effect will require additional investigation.

Second, our results did not identify differences in vocal activity suggesting that acoustic differences are not based on a general weakness of the homozygote pups. Homozygote and wild-type pups showed typical ontogenetic changes (e.g., initial increase than decline in calling rates) that have been described for rats (e.g., Allin and Banks 1972; Brudzynski et al. 1999; Brudzynski 2005; Winslow 2009; Zeskind et al. 2011).

Third, maximum lung pressures did also not differ between homozygote and wild-type animals. This suggests that the slower breathing rhythm in dt rats is not a manifestation of somatic weakness. Respiratory rhythm in homozygote animals was affected early during development and remained affected into adulthood. Despite the slower breathing rhythm in homozygote dt rats, the ratio of expiratory and inspiratory phases did not differ.

Eupnoeic breathing is characterized by three phases of the respiratory cycle: inspiration, post-inspiration (or passive expiration), and active (or late) expiration (Smith et al. 2013). In awake rats, the respiratory pattern is modulated by motor activities (Kabir et al. 2010). In general, respiratory patterns in mammals are generated in two medullary sites, the pre-Bötzinger complex and the more rostral parafacial respiratory group (Kolliker-Fuse nucleus) (e.g., Smith et al. 1991; Onimaru et al. 2006). Furthermore, respiratory responses elicited by mechanical or chemical perturbations is modulated by multiple brain sites (e.g., Dreschaj et al. 2003; Feldman et al. 2003), including the cerebellum (Xu and Frazier 1997, 2000). A complex neuronal network in the brainstem is also involved in vocal pattern generation (Yajima et al. 1980; Yajima and Hayashi 1983; Hage and Jürgens 2006), and in vocal-respiratory rhythms entrainment (Smotherman et al. 2006). Recent studies in two mice species have identified projections from laryngeal muscles in the motor cortex (Arriaga et al. 2012; Okobi et al. 2014). Accordingly, we propose two hypotheses for the pathobiology of the slowed breathing rates in homozygote dt rats with effects on vocal production. First, the cerebellar dysfunction in homozygote dt rat pups disturbs the development of a normal breathing rhythm. Respiratory patterns mature during the first 2 weeks of a rat's life, and the adult respiratory pattern is fully recognizable after P15 (Paton and Richter 1995; Dutschmann et al. 2009). It is possible that chronic pathological cerebellar output disturbs this maturation process. Alternatively or complementary, caytaxin deficiency exerts deleterious effects on non-cerebellar neuronal populations involved in regulation of the breathing rhythm. Caytaxin is a brain-specific member of the BNIP2 family, which is conserved across mammals, including rodents and humans (Sikora et al. 2012). Caytaxin is encoded by *Atcay*. Loss-of-function recessive mutations in *Atcay* cause a neurological syndrome in humans commonly referred to as Cayman ataxia and dystonia in several murine models, including jittery mice and the dt rat (LeDoux 2011). Caytain is widely expressed in sensorimotor structures of the brain, including cerebellum, brainstem, cerebral cortex, striatum, and thalamus (Xiao and LeDoux 2005; Buschdorf et al. 2006). Expression of caytaxin is developmentally regulated and appears to play an important role in neural differentiation (Xiao, LeDoux 2005; Hayakawa et al. 2007). Its role in maintaining normal neuronal function is not entirely clear (Buschdorf et al. 2006; Aoyama et al. 2009). It is conceivable that caytaxin deficiency in extra-cerebellar regions such as medullary respiratory pattern generators could be responsible for the slowed breathing cycle in the homozygote rats.

Breathing abnormalities found in association with cerebellar dysfunction have also been observed in other rodent models and humans. A mouse model of Leigh syndrome demonstrates periods of apnea and an aberrant response to hypoxia (Quintana et al. 2012). The model showed lesions in the dorsal brain stem vestibular nucleus, abnormal neuronal firing patterns in the pre-Bötzinger complex and lesions in the deep cerebellar fastigial nucleus. In humans, congenital central hypoventilation syndrome and the sudden infant death syndrome are associated with brainstem and cerebellar abnormalities (Harper 2000; Gaultier et al. 2004; Kumar et al. 2008).

Our description of altered breathing rhythms in a genetic dystonia model highlights the relevance and utility of using rodents for research into respiratory and phonatory difficulties (arrhythmic breathing; apnea; interruptions during speech production) that can be important clinical manifestations in patients with dystonia, Parkinson's disease and other movement disorders (Braun et al. 1995; Lagueny et al. 1995; Mehanna and Jankovic 2012). Dystonia may compromise respiration via dysfunction of the diaphragm, intercostal muscles or laryngeal muscles (laryngeal, cranio-cervical and trunk dystonia). The precise clinical parameters and mechanisms of respiratory and phonatory dysfunction in dystonia have not been well characterized and animal models offer the possibility of localizing defective neural circuits such as the olivocerebellar pathway or respiratory pattern generator. For example, specific expression of FOXP2 in cerebellar Purkinje cells improved USV in heterozygous FOXP2 p.R552H knock-in pups, a model system for the human disorder developmental verbal dyspraxia (Fujita-Jimbo and Momoi 2014).

## Conclusions

Our study highlights the value of a multifaceted quantitative approach to study vocal control using similar methods as in songbird models (Riede and Goller 2010). Unfortunately, numerous publications that include data on vocal differences in murine disease models simply ascribe acoustic variability to genotypes rather than attempting to identify sites of neuronal dysfunction in the central and/or peripheral nervous systems. The effects of hearing maturation and developmental transition of ingestive behaviors on vocal production are poorly characterized or largely ignored. Clearly, additional physiological studies related to the role of specific sensorimotor neural structures in controlling the vocal organ and respiratory system in model systems are required for advancements in this field. Moreover, these experiments must correlate lesions or recordings from the central nervous system with comprehensive and exacting peripheral assessments of breathing and phonation. With regard to the latter, we showed that some motor changes such as an altered breathing rhythm, may not be captured by traditional acoustic analysis.

## Conflict of Interests

None declared.
